# Novel use of endoscopic morcellation of a duodenal bulb polyp

**DOI:** 10.1016/j.vgie.2024.09.011

**Published:** 2024-09-11

**Authors:** Shae Patel, Savan Kabaria, Paul Leonor

**Affiliations:** Department of Gastroenterology, Loma Linda University, Loma Linda, California, USA

## Background

**Figure 1:**
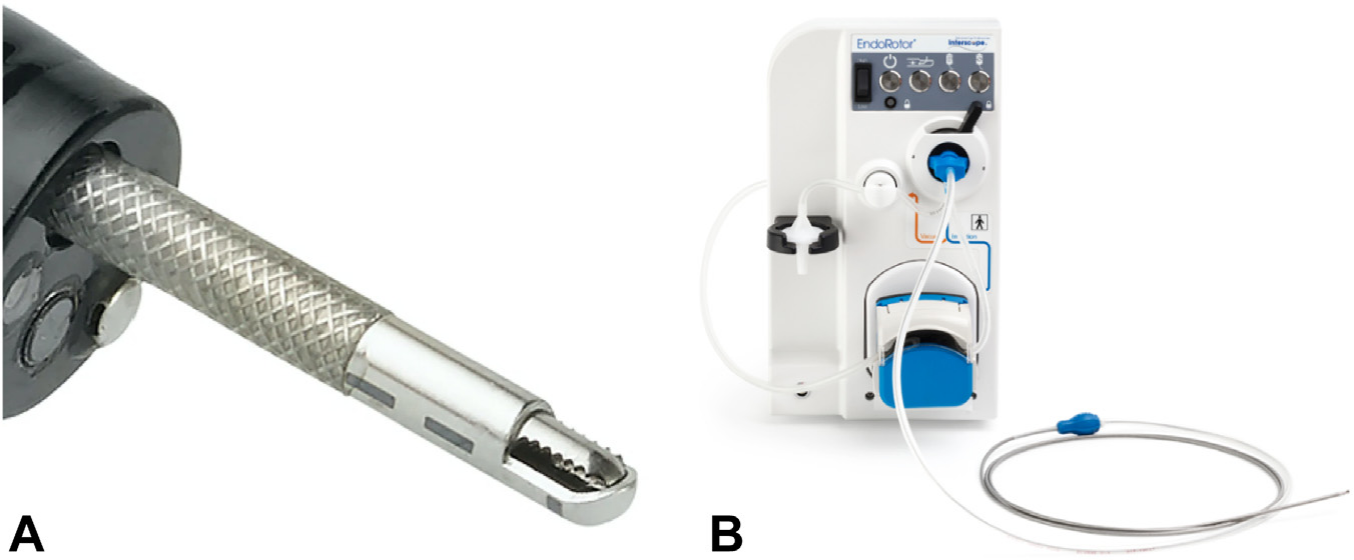
**A,** Endoscopic morcellator catheter (closeup). **B,** Morcellator console.

The conventional management of duodenal polyps poses challenges because of their anatomic location and potential adverse events. Typically, duodenal polyps are removed using a snare technique either via conventional or underwater EMR.^1^ However, large polyps in the proximal duodenal bulb can be difficult to adequately resect as the result of unstable endoscope positioning.^2^ This case report explores a novel approach by using endoscopic morcellation for the removal of a challenging duodenal bulb polyp (Fig. 1). This technique adds to the therapeutic options for the management of polyps that are difficult to endoscopically resect.

## Case presentation

**Figure 2:**
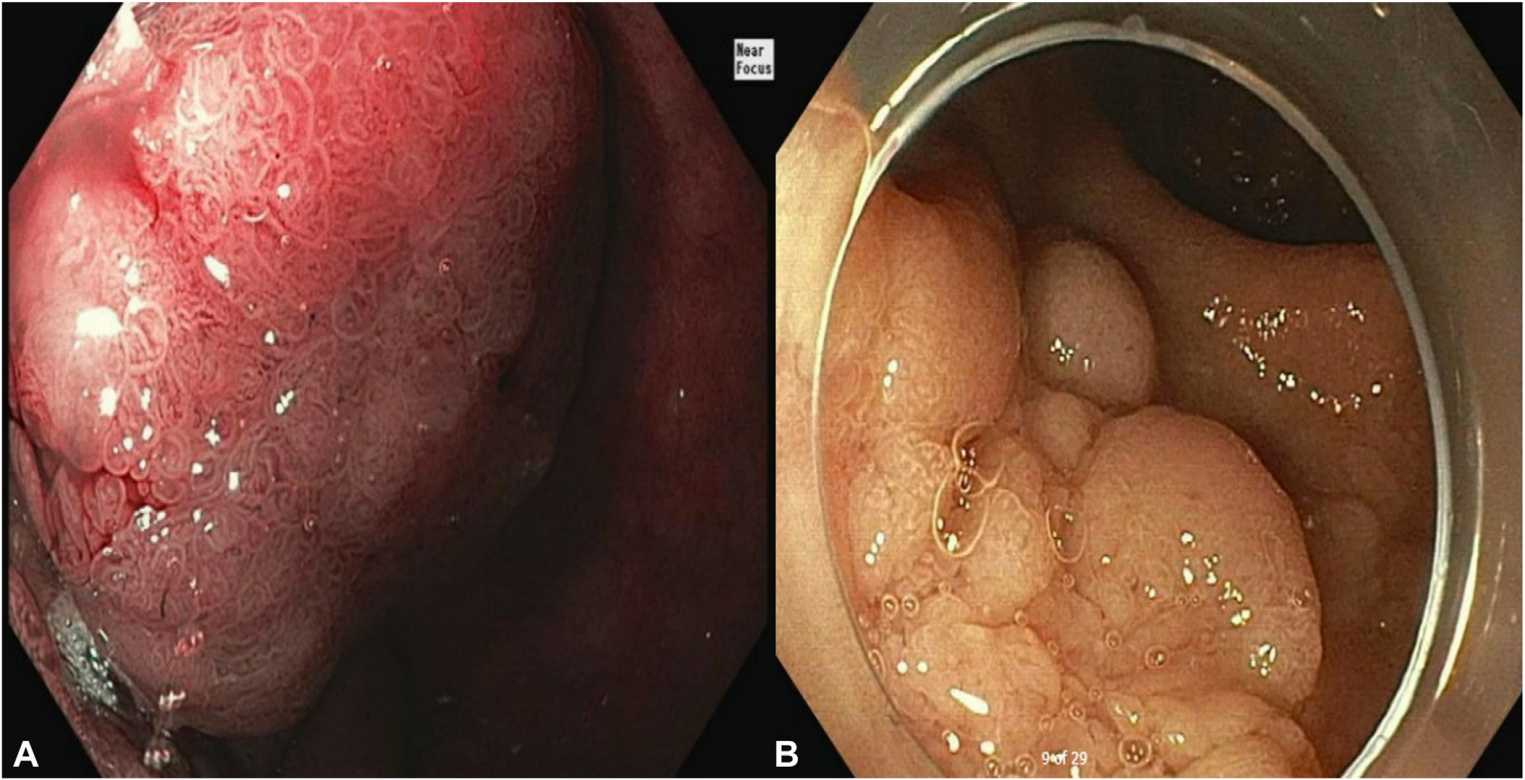
**A,** Proximal portion of polyp. **B,** Distal portion of polyp.

**Figure 3:**
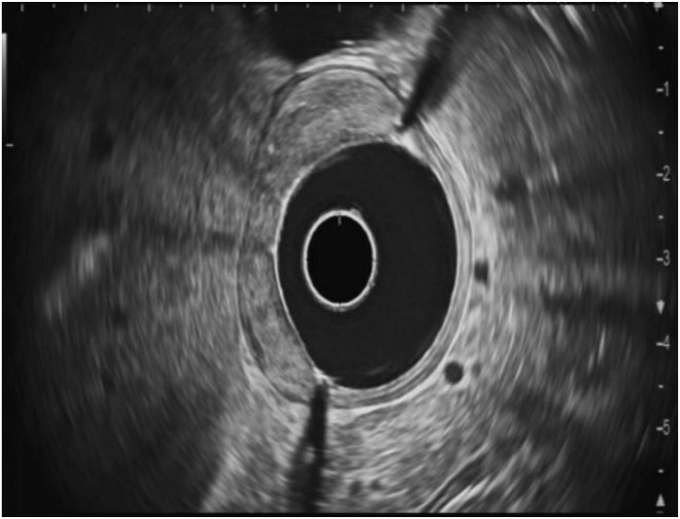
EUS showing no extension of the polyp into the submucosa or muscularis propia.

A 79-year-old woman with a history of congestive heart failure, paroxysmal atrial fibrillation, type 2 diabetes mellitus, breast cancer, cervical cancer, and iron deficiency anemia underwent an EGD to evaluate her anemia. She was found to have a large lobular duodenal bulb polyp just distal to the pylorus. The polyp involved approximately half the circumference of the duodenal bulb lumen, measuring approximately 4 cm in total diameter (Fig. 2). EUS of the polyp did not appear to show extension into the submucosa or involve the muscularis propia (Fig. 3). The initial attempt at conventional EMR was unsuccessful because of the unstable endoscope position and the location of the polyp extending to the superior aspects of the duodenal bulb. The pathology results of portions that were resected were positive for tubular adenoma. As a result of her medical comorbidities, the patient was deemed a high-risk surgical candidate for a Whipple procedure by the surgical oncology team. After discussion with the patient, the decision was made to attempt endoscopic morcellation resection of the polyp.

**Figure 4:**
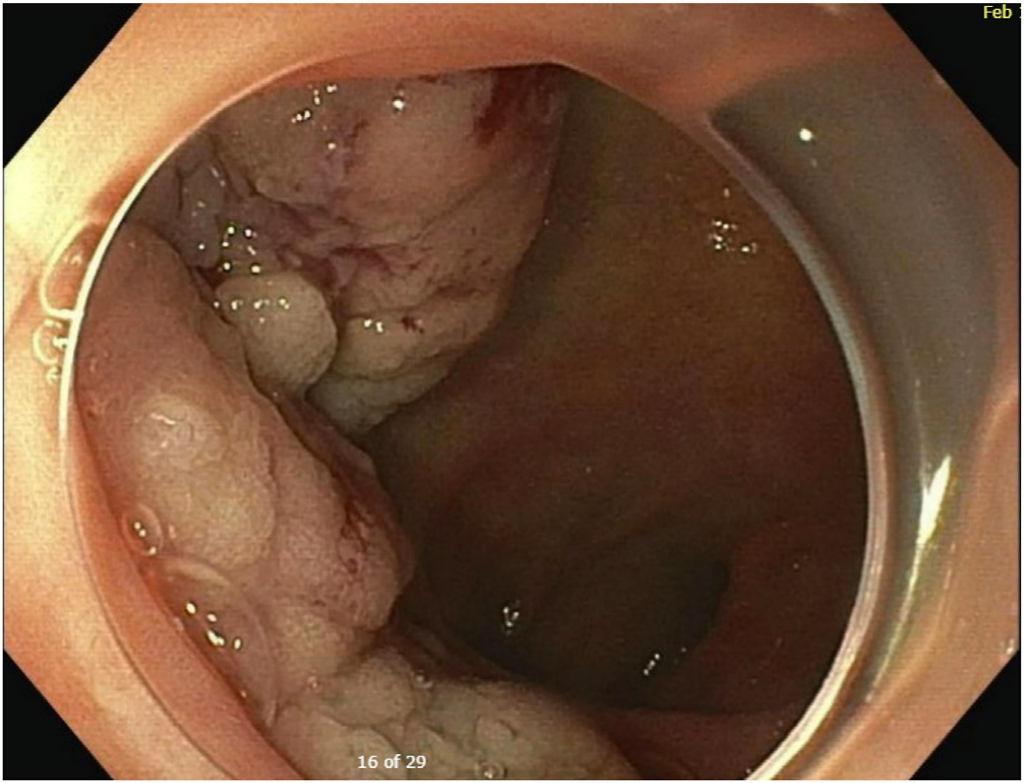
Blanching of polyp after 1:1000 epinephrine and saline injected.

**Figure 5:**
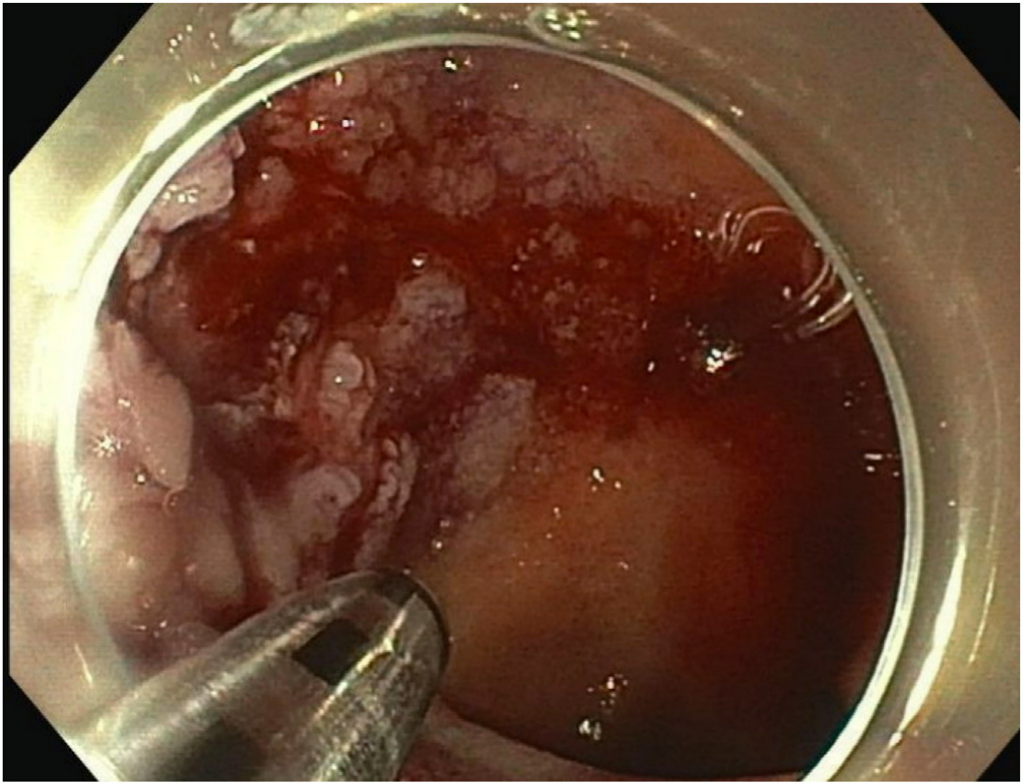
Use of endoscopic morcellator to resect the polyp.

**Figure 6:**
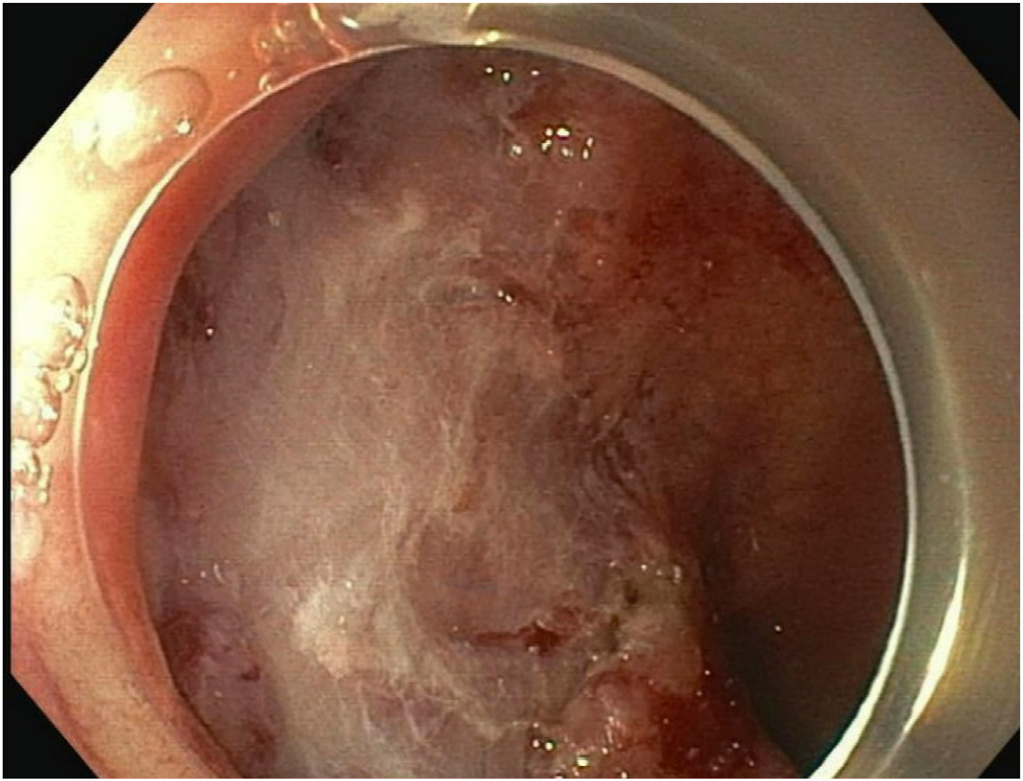
Complete resection of polyp with endoscopic morcellation.

Upon repeat EGD (Video 1, available online at www.videogie.org), the polyp was injected with a mixture of 1:10,000 epinephrine and saline with appropriate blanching (Fig. 4). The bulkier portions of the polyp were removed with a combination of hot and cold snare. The endoscopic morcellator was then used to resect the difficult portions of the polyp, particularly the scarred-down areas and the regions that were difficult to grasp with the snare (Fig. 5). The entire resected area was approximately 4 cm in diameter (Fig. 6). There was no evidence of perforation or active bleeding at the end of the procedure. Pathology results demonstrated tubular adenoma and incidental well-differentiated neuroendocrine tumor.

**Figure 7:**
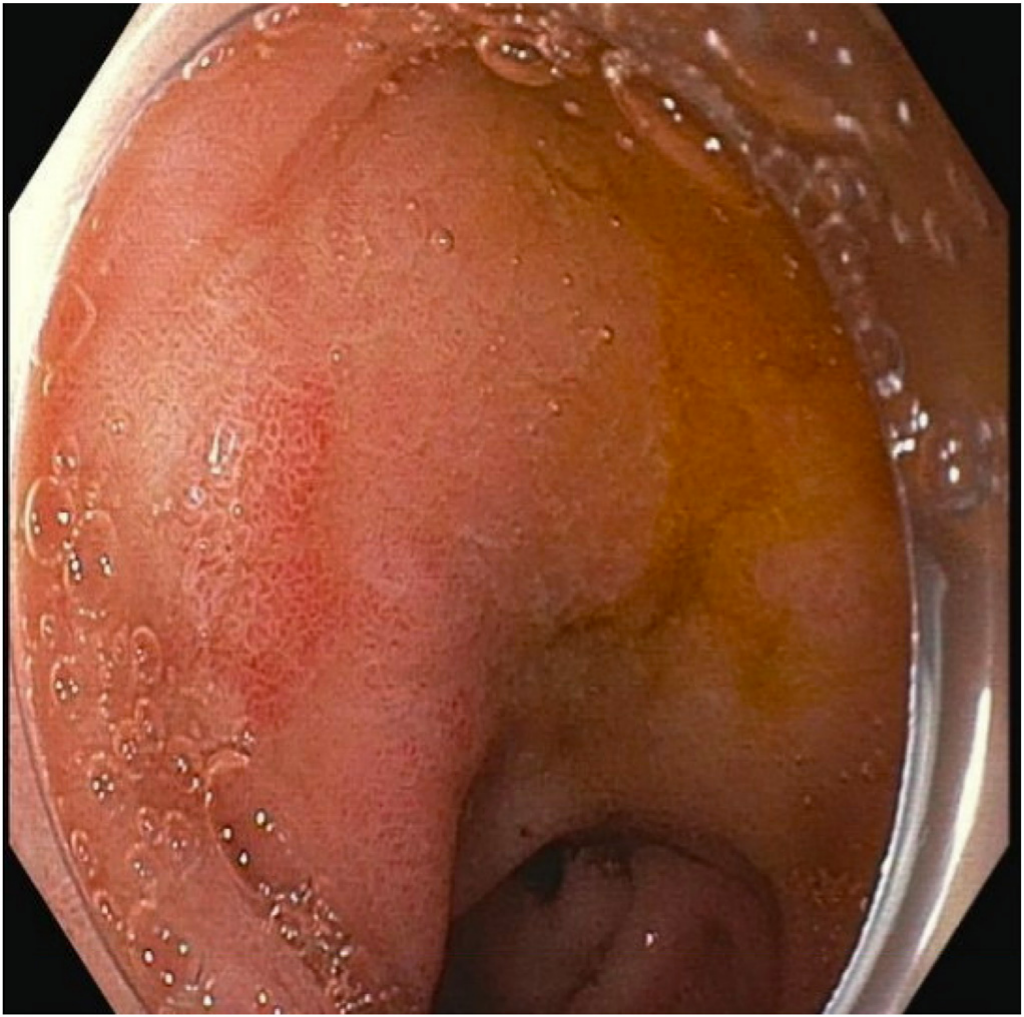
Mildly erythematous mucosa and reactive-appearing neoepithelium seen upon re-evaluation at 3 months.

Surveillance endoscopy performed 3 months later showed mildly erythematous mucosa at the site of resection with reactive-appearing neoepithelium without endoscopic evidence of recurrent or residual adenoma (Fig. 7). Random biopsies of the area did not show adenomatous tissue or evidence of residual or recurrent neuroendocrine tumor.

## Conclusions

We report a novel use of endoscopic morcellation to assist in removing a challenging duodenal bulb polyp. Endoscopic morcellation provides a new technique that is a safe and effective modality for the management of challenging duodenal polyp removal.

## Patient consent

The patient in this article has given written informed consent to publication of the case details.

## Disclosure

All authors disclosed no financial relationships.

## Supplementary data

Video 1 Endoscopic morcellation removal of a duodenal polyp.
